# Improvement of mechanical and antibacterial properties of porous nHA scaffolds by fluorinated graphene oxide

**DOI:** 10.1039/d2ra03854d

**Published:** 2022-09-07

**Authors:** Zexian Xu, Yali Li, Dian Xu, Li Li, Yaoxiang Xu, Liqiang Chen, Yanshan Liu, Jian Sun

**Affiliations:** The Affiliated Hospital of Qingdao University Qingdao China surgeonshan@outlook.com sunjianqdfy@qdu.edu.cn; School of Stomatology of Qingdao University Qingdao China; Dental Digital Medicine & 3D Printing Engineering Laboratory of Qingdao Qingdao China; Shandong Provincial Key Laboratory of Digital Medicine and Computer-Assisted Surgery Qingdao China

## Abstract

Nano-hydroxyapatite (nHA) is widely used as a bio-scaffold material due to its good bioactivity and biocompatibility. In this study, fluorinated graphene oxide (FG) was added to nHA to improve its poor formability, weak mechanical properties, undesirable antimicrobial activity and other disadvantages that affect its clinical application. FG was synthesized by a simple hydrothermal method. Novel porous composite scaffolds were prepared by adding different weight ratios (0.1 wt%, 0.5 wt% and 1 wt%) of FG to nHA using the 3D printing technique. The morphology, phase composition and mechanical properties of the composite scaffolds were characterized. In addition, the degradation performance of the composite scaffolds, antibacterial activity against *Staphylococcus aureus* and *Escherichia coli*, and cytocompatibility were also investigated. The results showed that the nHA/FG composite scaffold was successfully prepared with a uniform distribution of FG on the scaffold. The mechanical properties showed that the compression strength of the nHA/FG composite scaffold was significantly higher than that of the nHA scaffold (7.22 ± 1.43 MPa). The porosity of all composite scaffolds was above 70%. The addition of FG significantly improved the mechanical properties of the nHA scaffold without affecting the porosity of the scaffold. In addition, the 0.5 wt% nHA/FG scaffold exhibited satisfactory cytocompatibility and antibacterial properties. Therefore, the constructed nHA/FG composite scaffold can be considered as a novel antimicrobial bone substitute material with good application prospects.

## Abstract 2

Nano-hydroxyapatite (nHA) is widely used as a bio-scaffold material. In this study, fluorinated graphene oxide (FG) was added to nHA to improve its poor formability, weak mechanical properties and undesirable antimicrobial activity that affect its clinical application.

## Graphical abstract

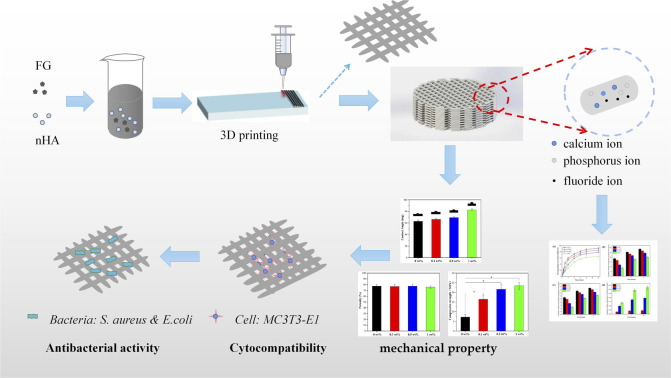

## Introduction

The repair and reconstruction of jaw bone defects caused by tumors, trauma, and congenital dysplasia diseases has always been difficult in clinical treatment.^1^ With the development of biomaterials, bone tissue engineering scaffolds have been widely used in the medical field, providing a new approach to achieving jaw bone regeneration.^2^ However, the present scaffolds have shortcomings such as weak mechanical properties and poor antibacterial effect, which lead to the failure of jaw bone regeneration.^3,4^ At present, for the preparation of composite scaffolds, researchers are mixing polymeric materials with nanomaterials by 3D printing to fully utilize the advantages of both substances and improve their performance.^5^

Hydroxyapatite (HA), the main inorganic component of natural bone, has good bioactivity and osteoinduction, which can be incorporated into new bone, promote osteoblast ossification and differentiation, as well as repair and replace damaged or traumatized bone tissue.^6^ However, its poor formability, weak compressive strength, and difficulty in degradation have limited its application in bone repair.^7^ By contrast, nano-hydroxyapatite (nHA) can effectively ameliorate these problems. The nHA has significantly higher surface area, porosity and densification, which can improve its mechanical properties.^8^ In addition, the nanoscale morphology has a positive effect on the proliferation and differentiation of osteoblasts, allowing bone regeneration and thus improving biocompatibility and osseointegration.^9^ Unfortunately, this feature may also lead to bacterial adhesion and biofilm formation.^10^ When such materials are implanted in bone defects, especially in bone defect caused by osteomyelitis, the surgery often fails due to bacterial infection. Bacterial infection can lead to hypersensitivity, inflammation, and necrosis of tissue in the implanted area. Some researchers have proposed mixing nHA with antibiotics (*e.g.*, amoxicillin, erythromycin, and minocycline) to treat associated infections.^11,12^ However, long-term use of these added antibiotics can lead to bacterial resistance and damage the mechanical properties of scaffolds. Meanwhile, the addition of nanomaterials such as graphene-based materials have been applied to improve the performance of scaffolds. For example, graphene oxide (GO) has abundant hydroxyl and carboxyl groups, which can promote osteoblastic differentiation and antibacterial activity in bone tissue engineering.^13^ When GO comes into contact with bacteria, its sharp edges violently cut the cell membrane, leading to the destruction of the cell membrane structure and the release of its intracellular contents, which further leads to the death of bacteria. Previous studies have reported that GO has certain effects on the proliferation, differentiation and adhesion of cells. HA/GO composites have good biocompatibility and biomechanical strength, and their elastic modulus can be well matched to human bone.^14^ Moreover, GO-containing composite scaffold can not only deactivate bacteria with impressive effects, but it also shows high osteoinductivity capacities.^15^

In recent years, studies have shown that GO nanosheets can absorb ions, molecules or complexes on their surface through different mechanisms (electrostatic, coordination bonds, *etc.*), and release them under certain conditions.^16,17^ Recently, some scholars have used GO and hydrofluoric acid as the main raw materials to prepare fluorinated graphene oxide (FG) by a hydrothermal reaction method. As a novel biomaterial, FG not only retains the advantages of GO, such as its two-dimensional structure and mechanical strength, but also distinguishes itself from other graphene derivatives by its unique C–F bond.^18^ FG has a significant stimulating effect on osteoblasts, and an appropriate concentration of FG promotes bone mineralization and formation.^19^ Moreover, FG can exert antibacterial effects by inhibiting the growth, reproduction and metabolism of bacteria and interfering with the formation of bacterial biofilms.^20^ Due to the addition of fluorine that confers biocompatibility, strong hydrophobicity, and low surface energy, and FG has potential applications in the biomedical field.^21–23^ Previous works have shown that FG has high antibacterial activity and can be used to design more effective graphene-based antibacterial agents.^24^ Xu *et al.* found that FG synthesized from GO has higher antibacterial activity than GO.^25^ In addition, FG can effectively improve the mechanical, tribological and antibacterial properties of glass ionomer cements, offering the possibility of dental material applications.^26^ However, the research on FG is still in its infancy, and little attention has been paid to the application of FG in the field of bone tissue engineering.

Based on these, nHA/FG porous composite scaffolds were prepared by 3D printing, and the composition, mechanical properties and antibacterial properties of the composite scaffolds were investigated to develop a novel bone tissue engineering scaffold material.

## Materials and methods

### Materials

nHA was purchased from Sigma-Aldrich, USA, with an average particle size of 100 nm. Graphene oxide (GO) was obtained from Pioneer Nanotechnology (Nanjing, China). Mouse embryo osteoblast precursor (MC3T3-E1) cells were provided by the Cell Room, School of Stomatology, Qingdao University. *Staphylococcus aureus* strains (*S. aureus*, ATCC 6538) and *Escherichia coli* strains (*E. coli*, ATCC 8739) were supplied by Haibo Biology Co., Ltd (Qingdao, China). All other reagents used were of analytical grade.

### Preparation of FG

According to a previous report,^26^ FG was prepared by hydrothermal reaction using GO and hydrofluoric acid as the main raw materials. Briefly, 100 mg of GO was dispersed in 80 mL of deionized water and the dispersion was ultrasonicated for one hour. Subsequently, 10 mL of concentrated nitric acid and hydrofluoric acid were added to the GO dispersion and stirred uniformly. The mixed dispersion was transferred into a Teflon-lined autoclave and heated at 180 °C for 12 hours. Finally, the obtained solution was heated directly at 50 °C in an oil bath to evaporate water and the FG powder was synthesized.

### Preparation of nHA/FG composite scaffolds

A series of nHA/FG porous composite scaffolds were prepared by mechanical mixing and 3D printing. First, FG was dispersed in 50 mL of absolute ethanol using ultrasound, and then a certain amount of nHA was added (the percentage of FG in the nHA/FG mixture were 0.1 wt%, 0.5 wt% and 1 wt%, respectively). After one hour of ultrasonication, the mixtures were put into the oven to completely evaporate the ethanol to obtain the well-mixed nHA/FG powder. Then, the slurry containing nHA/FG powder was transferred to the syringe of the 3D bioprinter (Wuwei Technology Co., Ltd, Qingdao, China) and the air bubbles were expelled. The scaffolds were designed with computer-aided design software and printed under the following specific parameters: printing speed 4 mm min^−1^; print head diameter 100 μm; and printing pitch 300 μm. Finally, the printed porous composite scaffolds were dried at room temperature for 24 hours, and then sintered at 1100 °C in a muffle furnace at the rate of 2 °C min^−1^. After sintering for 2 hours, the experimental scaffolds were obtained and sterilized for use.

The composite scaffolds with different FG contents of 0 wt%, 0.1 wt%, 0.5 wt% and 1 wt% were denoted as nHA (0 wt%), nHA/FG (0.1 wt%), nHA/FG (0.5 wt%) and nHA/FG (1 wt%), respectively. nHA (0 wt%) was the control group and other groups were the experimental groups.

### Characterization of synthesized FG and nHA/FG composite scaffolds

Transmission electron microscopy (TEM, JEM-2100F, JEOL) and atomic force microscopy (AFM, Dimension ICON, Bruker) were used to observe the morphology and thickness of the FG. The size distribution of FG was measured through TEM images using ImageJ software. Chemical composition and chemical bonding states were characterized by X-ray photoelectron spectroscopy (XPS, ESCALAB 250XI, Thermo Scientific) and X-ray diffraction (XRD, X'Pert Pro MPD, PANalytical). The structure of FG was measured by Raman spectra (Lab RAM HR800), the excitation wavelength was *λ* = 532 nm, and the scanning range was 500 to 4000 cm^−1^. The characteristic bands were observed with Fourier transform infrared spectrometer (FTIR, Nicolet 380 IR, Thermo Scientific) in the wave number range of 500 to 4000 cm^−1^.

The micro-morphology of composite scaffolds were observed by SEM (JSM 6701F, JEOL) equipped with an energy dispersive X-ray analyzer (EDX). The structures and compositions of nHA scaffold and nHA/FG composite scaffolds were analyzed by FTIR, Raman, XRD and XPS.

### Measurement of the contact angle, porosity and compressive strength

The water contact angle of each group of scaffolds was measured with an SL200B contact angle tester and analyzed for hydrophilicity. Nearly 5 μL of distilled water was dropped on the sample surface using a quantitative system, and the contact angle was recorded after two seconds. The test was repeated five times.

The porosity (*P*) of the scaffold was measured by the liquid displacement method,^27^ using absolute ethanol as the moving fluid into the scaffold pores initial volume of absolute ethanol was recorded as *V*_1_. The scaffold was placed in the graduated cylinder of absolute ethanol solution and immersed for 5 min. The scaffold was completely saturated by the absolute ethanol solution, at which point the total volume was recorded as *V*_2_. And *V*_3_ was the volume of absolute ethanol remaining after removal of the scaffold. The porosity of the scaffold was estimated using the following equation:1
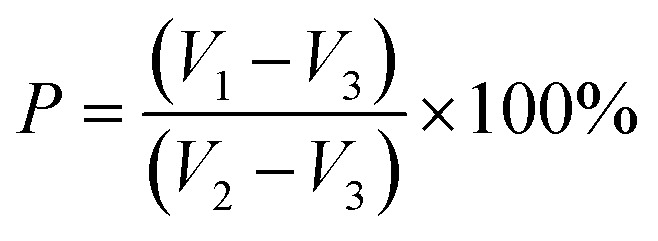


The compressive strength of each group of scaffolds was measured using an electronic universal testing machine. Samples were made into cylinders with *d* = 10 mm and *h* = 5 mm and the compression properties of the samples were tested at a rate of 1 mm min^−1^. Five samples were tested in each group.

### Investigation of the degradation performance and ion release

Five samples of each group were prepared to investigate the degradation performance. Each set of scaffolds was weighed after drying and recorded as *M*_1_. After weighing, the scaffold was immersed in 10 mL of phosphate buffer solution (PBS, pH = 7.4) and placed in a 37 °C incubator for degradation for six months. The PBS was changed at 1, 2, 4, 8, 12, 16 and 24 weeks. Then the scaffold was washed, dried and weighed, denoted as *M*_2_. The degradation rate (DR) at each time point was calculated and the degradation rate curve was drawn. The degradation rate of the scaffold was estimated using the following equation:^28^2
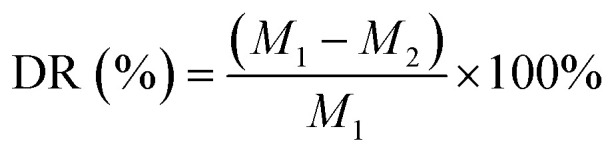


Each sample was immersed in 10 mL of deionized water for 1, 3 and 6 months, respectively. At the given time points, the extracts were replaced of the fresh 10 mL deionized water and detected. Calcium and phosphorus ion release from scaffolds were measured by inductively coupled plasma spectrometer (ICP-AES, Agilent 725-ES). The fluoride ion concentration was determined by fluoride ion-selective electrode connected to an expandable ion analyzer. The cumulative release concentrations were obtained by adding up the concentration at each time point. Five parallel samples were tested in each group.

### Cytocompatibility and blood compatibility

MC3T3-E1 cells were used to assess the cytocompatibility of the scaffolds. Four sets of scaffolds were placed in 24-well plates on the ultra-clean bench and placed under UV light for 24 hours. Then α-DMEM medium was dripped into each well to wet the scaffolds. The 24-well plate was placed in a cell incubator containing 5% CO_2_ for 24 h. Cell suspensions at a concentration of 2 × 10^4^ cells per mL were inoculated into 24-well plates at a volume of 100 μL per well. 1, 3, and 5 days later, 10 μL of Cell Counting Kit-8 (CCK-8) was added to each well at each time point, and the absorbance of each well at 450 nm was measured using an enzyme marker. The cell proliferation were visualized by FDA/PI staining after incubation for 1 and 5 days and by fluorescence microscopy.

The blood compatibility of the material was evaluated by calculating the hemolysis rate according to ISO 10993-4 standard.^29^ First, 4 mL of the venous blood of healthy volunteer was mixed with 5 mL of normal saline to obtain diluted blood for use. After that, the scaffold materials were sterilized and placed in a centrifuge tube filled with 10 mL of physiological saline. 10 mL of physiological saline was taken as a negative control group, and 10 mL of deionized water was used as a positive control group. Then add 0.2 mL of diluted blood to each group of centrifuge tubes, mix well, and incubate at 37 °C for 1 h. Finally, each group of centrifuge tubes were centrifuged at 3000 rpm for 5 min, and the supernatant was added to the 96-well plate. Each tube was set with six duplicate wells. The optical density (OD) value at the absorption wavelength of 545 nm was detected by a enzyme marker. The hemolysis rate of the scaffold was calculated according to the formula:3
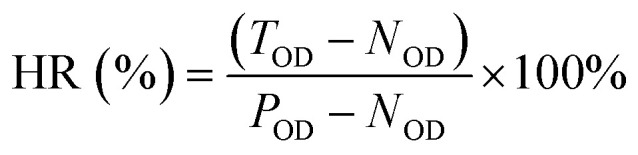
HR – hemolysis rate; *T*_OD_ – the OD value of test samples; *P*_OD_ – the OD value of positive control group; *N*_OD_ – the OD value of negative control group.

### Antibacterial activity

The bacterial strains used in this study were the representative Gram-positive bacterium *S. aureus* and the Gram-negative bacterium *E. coli*. nHA/FG composite scaffolds were evaluated for antibacterial activity by the colony counting method. Briefly, bacterial strains were picked with an inoculation loop and placed in Luria Bertani (LB) liquid medium, and the concentration of bacterial strains was adjusted to 10^8^ CFU per mL. Then the bacteria, LB liquid medium, and scaffolds were mixed and incubated in a constant-temperature incubator for 24 hours. Subsequently, the scaffolds were removed, washed several times with PBS solution, and placed in sterile centrifuge tubes containing sterile LB liquid medium. Afterwards, the tubes were shaken for 5 min to obtain bacteria adhering to the surface of the scaffolds. After gradient dilution of the obtained bacterial solutions, 100 μL of each dilution was inoculated onto LB agar plates and evenly coated. The total number of colonies was counted after 24 hours of incubation. The scaffold-free group was used as a blank control group. The experiment was repeated three times for each group. AR was calculated as following equation:^30^4
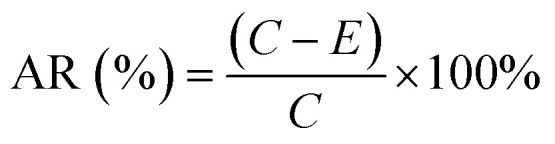
AR – antibacterial rate; *C* – average colony number of blank control group; *E* – average colony number of scaffolds in each group.

### Statistical analysis

Statistical analysis was performed by SPSS 23.0 software. All representative data were expressed as *x* ± *s*. One-way analysis of variance (ANOVA) and Tukey's honest significant difference (HSD) test were used, and values of *P* < 0.05 were considered statistically significant.

## Results

### Characterization of FG

**Fig. 1:**
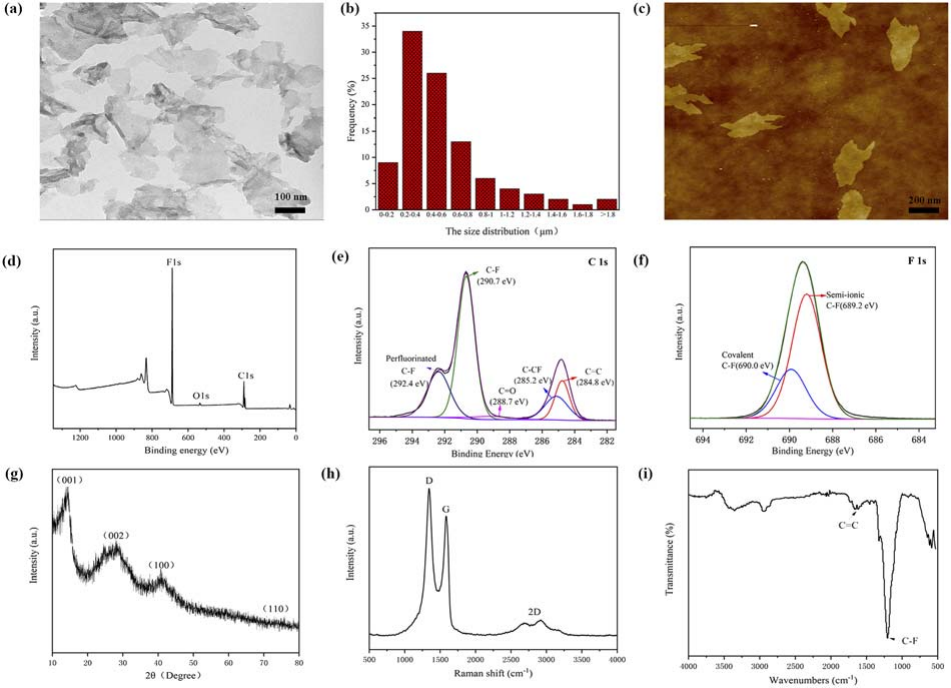
Characterization of FG: (a) TEM image, (b) size distribution, (c) AFM topography, (d) XPS survey spectrum, (e) C 1s XPS spectrum, (f) F 1s XPS spectrum, (g) XRD pattern, (h) Raman spectrum and (i) FTIR spectrum.

A large number of FG nanosheets can be clearly seen from the TEM image (Fig. 1a), and FG shows a transparent nanostructure with dimensions of about 0.2–2 μm (Fig. 1b) and some nanosheets have wrinkles on the edges. It can also be observed from the figure that there are some slightly darker parts, which is caused by the stacking and overlapping of the FG nanosheets. The AFM image of the FG (Fig. 1c) shows its lateral dimensions of about a few hundred nanometers. This result is consistent with the test results of TEM. The investigated XPS spectra (Fig. 1d) clearly show the presence of the elements C, O and F with binding energies of about 289 eV, 535 eV and 688.6 eV, respectively. The C 1s spectrum of FG shows the presence of carbon bonds (Fig. 1e), including C=C, C=O, C–F and C–F_2_.^31^ As seen from Fig. 1f, the F 1s spectrum consists of two peaks at 689.2 eV and 690.0 eV, corresponding to the semi-ionic C–F bond and the covalent C–F bond, respectively. The XRD pattern of FG (Fig. 1g) exhibits four peaks near 12.7°, 26.1°, 40.9° and 74.2°, corresponding to diffraction from the (001), (002), (100) and (110) planes, respectively. All peaks are in general agreement with the values of the standard JCPDS-ICDD pattern (PDF no. 41-1487).^32^ The characteristic diffraction peak of graphene (002) crystal plane appears at the 2*θ* degrees of 26.1°. The obvious diffraction peaks of FG (001) and (100) crystal plane indicate the existence of the hexagonal crystal plane with high fluorine content.^33^ As shown in Fig. 1h, the Raman spectra show two strong peaks in FG, namely the D and G peaks located around 1345 cm^−1^ and 1593 cm^−1^ respectively, and a weak 2D peak located at 2800 cm^−1^. The G and 2D peaks are the characteristic peaks of graphene, which are related to the in-plane vibration of sp^2^ carbon atoms.^34^ The appearance of the D peak proves that the atoms in FG are starting to appear disordered. This may be caused by the substitution of oxygen atoms by a large number of fluorine atoms.^35^ Therefore, it can be concluded that the FG exhibits the distinct characteristic peaks of graphene, while the ordered structure of the FG is disrupted. The FTIR spectroscopy (Fig. 1i) shows that a characteristic peak of C–F covalent bonding at 1202 cm^−1^, which indicates that most of the fluorine atoms have replaced oxygen atoms and successfully bounded to C atoms.^36^ The C=C absorption peak at 1620 cm^−1^ showed that FG still maintains part of the six-membered ring of sp^2^ structure. And the vibration peak at 3440 cm^−1^ is the O–H bond peak, indicating that some hydroxyl groups may not be removed.

### Characterization of prepared nHA/FG composite scaffolds

#### SEM

**Fig. 2:**
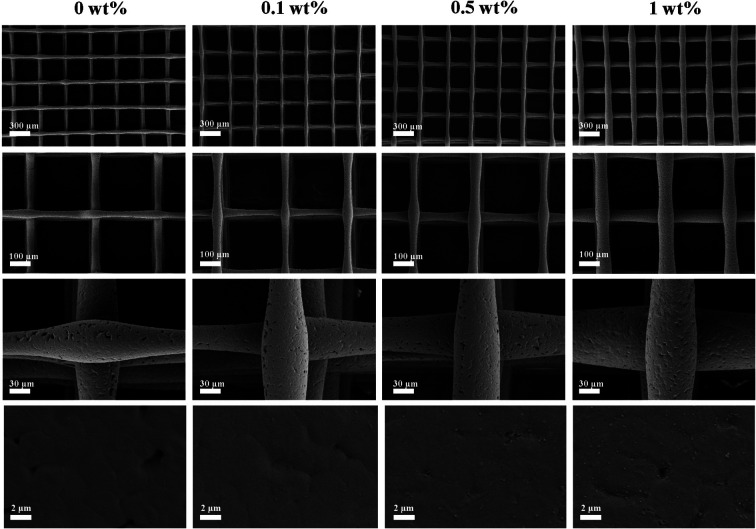
SEM images of the composite scaffolds at different magnifications.

Fig. 2 shows SEM scans of porous scaffolds with FG contents of 0 wt%, 0.1 wt%, 0.5 wt% and 1 wt%, respectively. From the SEM images, it can be seen that these scaffolds are regularly layered structures; and the surface of the scaffolds is porous three-dimensional network structure, forming a highly porous connection structure with a pore size of about 300 μm, which is conducive to the adhesion and growth of cells.^37^ Meanwhile, with the increase of FG concentration, the filament diameter was gradually uniform. Moreover, nanosheets on the composite scaffold surface were observed from high magnification.

#### Elemental analysis and distribution

**Fig. 3:**
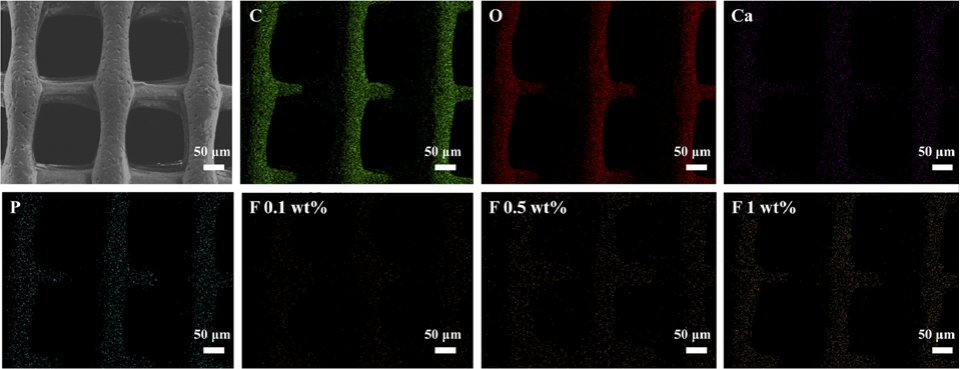
Elemental analysis and distribution of composite scaffolds.

Fig. 3 shows the elemental composition of the nHA/FG (0.5 wt%) composite scaffold and its distribution. It can be seen that the elements C, O, Ca, P and F are uniformly dispersed in the scaffolds, which may be due to the adequate ultrasonic dispersion of the hybrid slurry prior to 3D printing. In addition, the presence of these elements confirms the chemical composition of nHA and FG.

#### XPS, XRD, Raman and FTIR analysis

**Fig. 4:**
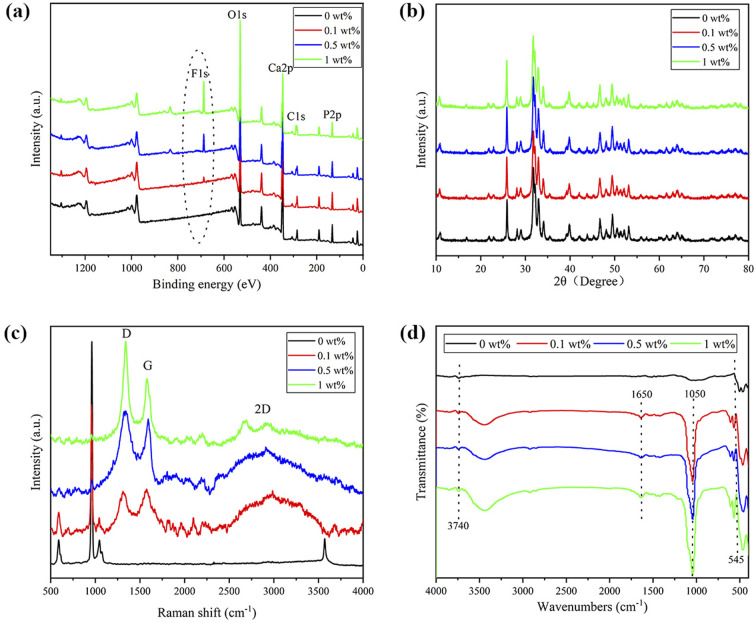
Characterization of nHA and nHA/FG composite scaffolds: (a) survey XPS spectra, (b) XRD pattern, (c) Raman spectrum and (d) FTIR spectrum.

The surface elements of nHA and nHA/FG composite scaffolds were obtained by XPS analysis. As shown in Fig. 4a, elements of C, O, Ca and P were detected in the nHA scaffold with binding energies of approximately 289 eV, 535 eV, 347 eV and 133 eV, respectively. In contrast to the nHA scaffold, element F was observed in the nHA/FG composite scaffold, which proved that FG was successfully bound.^38^ The peak corresponding to the F element became significantly higher as the FG ratio increased. This is consistent with the results of EDS.

Fig. 4b shows the XRD patterns of nHA and nHA/FG composite scaffolds. As shown, the nHA scaffold has intense diffraction peaks at 26.67°, 31.81° and 40.30°. Since FG is a weakly crystalline substance with less content, the shape and position of the diffraction peaks of the nHA/FG composite scaffold are similar to those of the nHA scaffold, and there are almost no diffraction peaks of FG in the XRD patterns.

Fig. 4c shows the Raman spectra of the nHA and nHA/FG composite scaffolds. It can be seen that the characteristic peaks at 1336 cm^−1^ and 1574 cm^−1^ in the groups of nHA/FG composite scaffolds correspond to the D peak and the G peak of FG, respectively. In addition, the nHA/FG composite scaffold has characteristic peaks near 589 cm^−1^, 959 cm^−1^ and 1061 cm^−1^, which are all characteristic peaks of nHA.^39^ The peaks at 589 cm^−1^ and 959 cm^−1^ represent the symmetrical bending vibration and asymmetrical bending vibrations of PO_4_^3−^ group. The nHA/FG composite scaffolds have the corresponding characteristic peak value on the nHA scaffolds and the characteristic peak value of the FG. It shows that FG and nHA materials are well combined.

Fig. 4d shows the FTIR pattern of nHA and nHA/FG composite scaffolds. It can be seen that the characteristic absorption peaks of PO_4_^3−^ are detected at 962 cm^−1^ and 1037 cm^−1^, while the absorption peaks of H_2_O are detected at 1626 cm^−1^ and 3430 cm^−1^. The characteristic absorption peak intensity of PO_4_^3−^ of the nHA/FG composite scaffold is obviously weakened due to the adsorption of FG on the surface of nHA. The stretching vibration bands of the C–F appearing at 1650 cm^−1^ and 1050 cm^−1^ belong to the characteristic absorption peaks of FG.^36^ The FTIR spectrum of the nHA/FG composite scaffold has similar parts to the FTIR spectrum of the nHA scaffold and the FG. However, with the increase of FG, the C–F peak at 1000–1250 cm^−1^ gradually becomes wider and stronger, and its peak position shifts laterally, which is consistent with the previous literature description.^40^

### Contact angle, porosity and compressive strength

**Fig. 5:**
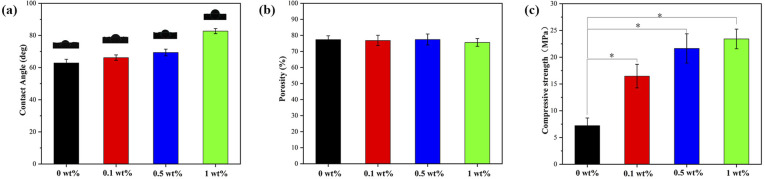
The mechanical properties of nHA and nHA/FG composite scaffolds: (a) the contact angle, (b) the porosity and (c) the compressive strength.

The results of contact angle (Fig. 5a) indicate that the nHA scaffold is highly hydrophilic, which facilitates cell adhesion and growth. In addition, the hydrophilicity of the composite scaffold decreased with the increase of FG addition. As can be seen in Fig. 5b, the porosity of all composite scaffolds is above 70%, and there is no significant difference between the groups. From Fig. 5c, it can be seen that the compressive strength of the composite scaffold gradually increases with the addition of FG. The highest compressive strength of 1 wt% group is 23.42 ± 1.84 MPa, while that of the lowest nHA group is only 7.22 ± 1.43 MPa.

### The degradation performance and ion release

**Fig. 6:**
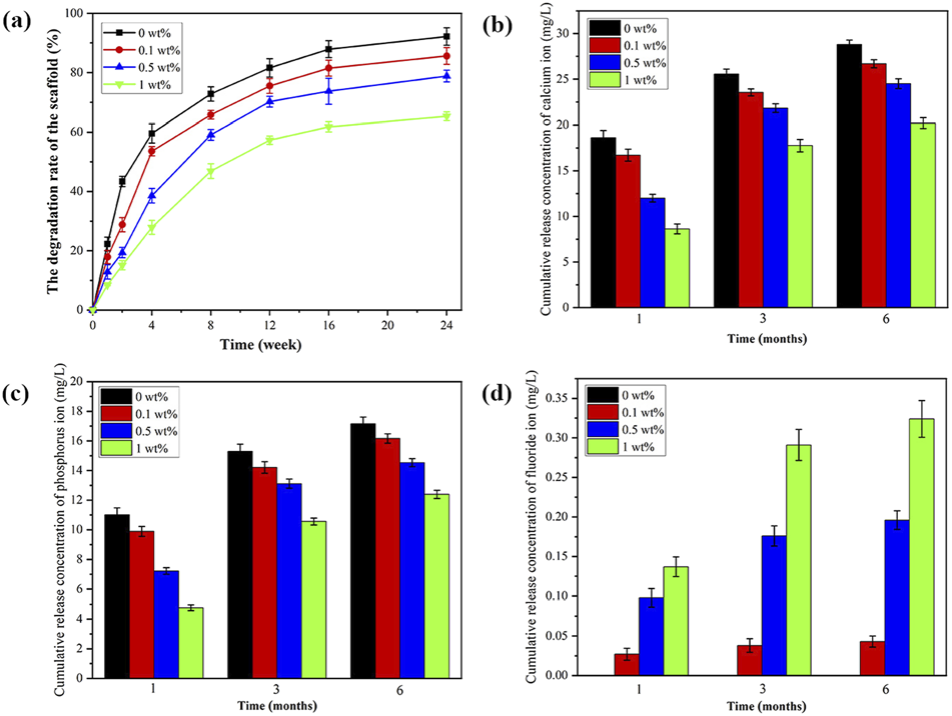
(a) The degradation, (b) the cumulative release of Ca ions, (c) the cumulative release of P ions and (d) the cumulative release of F ions of nHA and nHA/FG composite scaffolds.

As shown in Fig. 6a, the degradation of scaffold materials in each group increased with time, and the degradation rate exhibited a rapid degradation in the initial phase (within the first four weeks), followed by a slow and sustained degradation. At the 4th week, the degradation rates of 0 wt%, 0.1 wt%, 0.5 wt% and 1 wt% group were (59.62 ± 3.23)%, (53.64 ± 1.54)%, (38.71 ± 2.45)% and (27.92 ± 2.34)%, respectively. At the 12th week, the scaffold materials began to deform slightly, and the texture became brittle. At this time, the degradation rate of the 0 wt% group was (81.67 ± 3.13)%, and the 1 wt% group was (57.32 ± 1.40)%.

The release amounts of Ca and P ions from scaffolds in each group are shown in Fig. 6b and c. In the first month, the amounts of Ca and P ions released in each group were relatively large. With the addition of FG, the amounts of Ca and P ions gradually decreased. Within the same group, the amounts of Ca and P ions increased with time. Fig. 6d shows the cumulative release amounts of F ions. The nHA scaffolds do not release any F ions. Different from the release amounts of Ca and P ions, the release amounts of F ions gradually increased with the addition of FG. Ca, P and F ions are gradually released from the cracks and pores of the scaffold material, indicating that the release of ions has an important relationship with the degradation performance of the material.^41^ Although the degradation rate of 1 wt% group was slower, the total content of F in the scaffolds was higher. Therefore, the release amounts of Ca, P and F ions also depend on their total content in the scaffold.

### Cytocompatibility and blood compatibility

**Fig. 7:**
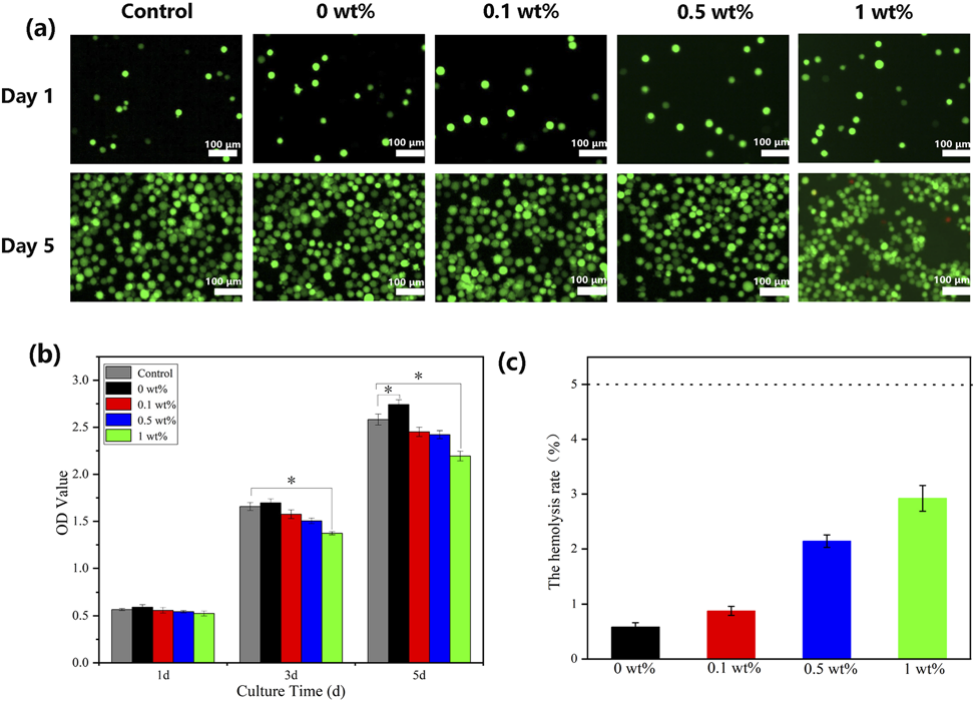
(a) Fluorescence microscopy images of FDA/PI staining, (b) CCK-8 assay results for MC3T3-E1 cells on composite scaffolds and (c) the hemolysis rate of composite scaffolds.

Fluorescence microscopy images and the results of CCK-8 (Fig. 7a and b) show that MC3T3-E1 cells were able to grow and proliferate in all four groups of scaffolds, and the number of cells increased with time (*P* < 0.05). This is due to the good porosity and roughness of the fabricated scaffolds, which provides a suitable environment for cell growth and proliferation.^37^ On the first day, there was no significant difference in cytocompatibility between the experimental group and the blank control group. However, on the 3rd and 5th days of culture, the nHA/FG composite scaffold had some inhibitory effect on the cells after the addition of FG. Cell growth and proliferation were slightly inhibited when the content of FG reached 1 wt% (*P* < 0.05). This indicates that the nHA/FG composite scaffold with low FG content has good biocompatibility, while the composite scaffold with high FG content has a certain degree of cytotoxicity.

According to the experimental evaluation standard,^30^ the *P*_OD_ should be 0.8 ± 0.3, and the *N*_OD_ should not be higher than 0.03. If the hemolysis rate of the material is less than 5%, it means that the material meets the requirements of the experiment for medical materials. In this experiment, the average *N*_OD_ was 0.007, and the average *P*_OD_ was 0.691, both met the experimental standard. The upper layer of both experimental groups and the negative control group were clear colorless liquid, and the lower layer of them were red blood cells sediment. The positive control group had hemolysis, and the color of the liquid was red. As shown in Fig. 7b, the hemolysis rate of test scaffolds were less than 5%, indicating that the scaffolds meet the requirements of the hemolysis test for medical materials.

### Antibacterial activity

**Fig. 8:**
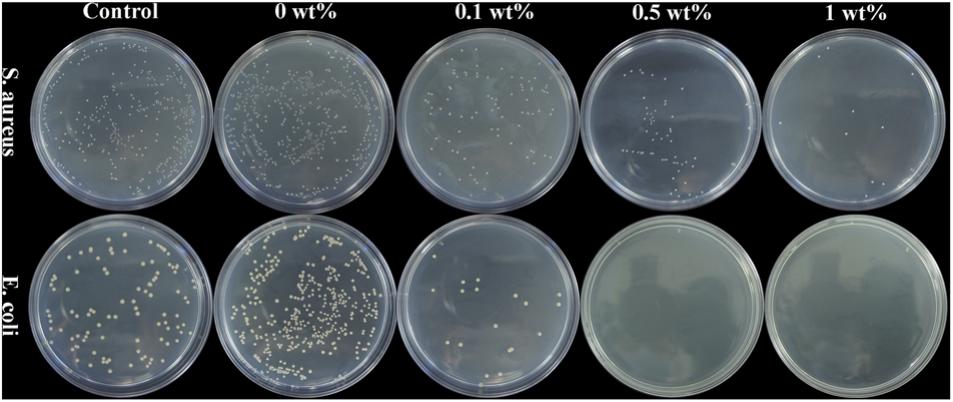
*S. aureus* and *E. coli* colonies on agar of blank control group, nHA (0 wt%), nHA/FG (0.1 wt%), nHA/FG (0.5 wt%) and nHA/FG (1 wt%).

**Fig. 9:**
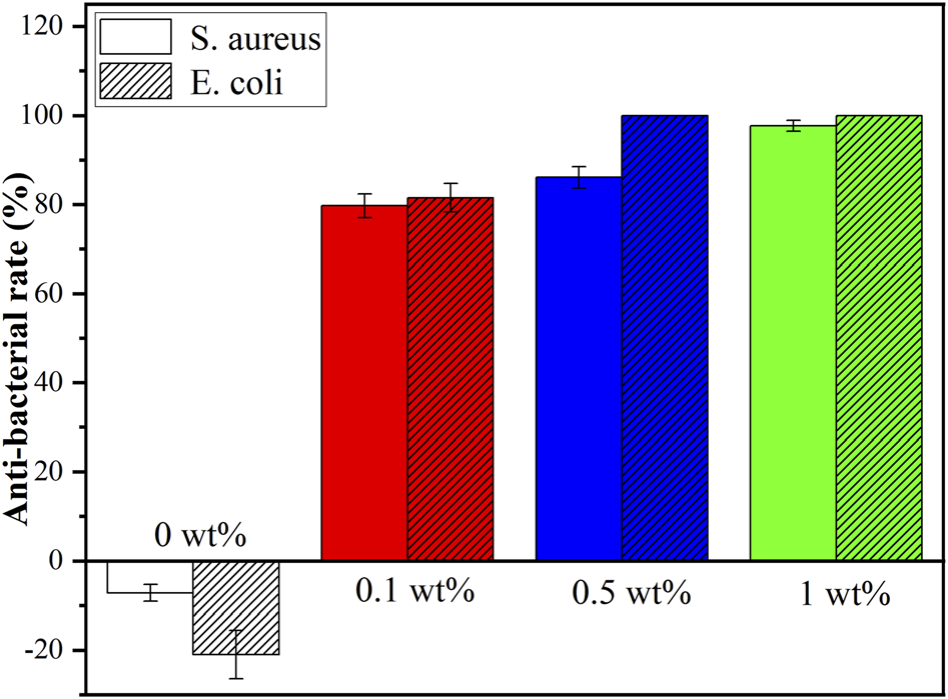
Antibacterial rate of the composite scaffolds.

Fig. 8 shows the colonization of *S. aureus* and *E. coli* on agar of blank control, nHA (0 wt%), nHA/FG (0.1 wt%), nHA/FG (0.5 wt%) and nHA/FG (1 wt%). The pure nHA scaffold promoted bacterial growth compared to the blank control group. All groups containing FG had some antibacterial properties. It can be seen that the number of colonies was reduced due to the addition of FG. In addition, the nHA/FG composite scaffold showed significantly stronger antibacterial activity against *E. coli* than against *S. aureus* (Fig. 9). It was proved that FG has antibacterial properties and it can be dispersed uniformly in the material as an antibacterial material.

## Discussion

In recent years, composite scaffolds using various advanced biomaterials have been widely used in the treatment of jaw defects. Ideally, composite scaffolds for bone tissue engineering should not only have good biosafety and osteogenic properties, but also have good antibacterial properties which help prevent bone infection after scaffold implantation. In addition, composite scaffolds should have some extent of mechanical properties and high porosity to ensure cell bioactivity, vascular formation, nutrient supply and waste removal.^42^ As an innovative material processing method, 3D printing technology can be more accurate in controlling the internal microstructure, the aperture size and the gross shape of the scaffold, which is more conducive for the personalized repair of bone defects in the clinical treatment.

In the experiment, FG and nHA were constructed into nHA/FG composite scaffolds through the 3D printing method. FG loaded into the scaffold plays a part in unique biological characteristics and good antibacterial performance. SEM showed that the morphology of nHA scaffold before and after FG loading did not change much, which was still staggered pore structure. With the increase of the amount of FG, the number of FG in the surface of the scaffold increased gradually. It is worth noting that the addition of FG did not significantly change the wire diameter of the composite scaffold at the beginning, but with the increase of the content of FG loaded, the wire diameter of the scaffold gradually increased. It may be deduced that the addition of FG increases the electrical conductivity of the printing, thus affecting the wire diameter of the composite scaffold. EDS, XPS, and Raman results all prove that FG and nHA materials are well combined. Moreover, the FTIR pattern inferred that FG and nHA may be effectively combined with each other by forming hydrogen bonds.

Porous scaffold materials for bone defect repair should have certain porosity, contact angle and compressive strength. According to relevant literature,^28^ the porosity of natural cancellous bone ranges from 30% to 90%, and the high porosity and interconnected bone scaffolds are conducive to cell adhesion and growth at the later stage of implantation in human tissues. In this study, the pores of the scaffolds were individually designed and printed. Through SEM images, relatively uniform pores and the structure in which the pores were interconnected could be observed. And the porosity was distributed between 77–79%, which is in accordance with the general requirements of bone tissue engineering scaffolds. In addition, the hydrophilicity and hydrophobicity of the scaffolds surface are considered to be important preconditions for the adhesion and growth of bone tissue cell. Typically, hydrophilic scaffold materials could promote cell adhesion and growth. The hydrophilicity of nHA/FG composite scaffolds were reduced with the increase of FG addition. This is related to the hydrophobicity of the functional groups on FG. However, composite scaffolds containing 1 wt% of FG is still less than 90°, indicating that this material is hydrophilic and promotes cell adhesion. Studies have shown that less hydrophilic scaffolds are more resistant to bacterial adhesion.^43^ This also explains the good antibacterial effect of the composite scaffold of the 1 wt% group. The compressive strength of the composite scaffold tended to increase markedly according to FG content. This may be due to the large number of modifiable functional groups on the surface of FG, which can bind to nHA through van der Waals forces and other chemical bonds.^28^ It plays a crucial role in stabilizing the material structure and enhancing the mechanical properties.

*In vitro* degradation of scaffolds is an important part of evaluating the physicochemical properties of scaffolds. Under physiological situation, the regeneration and repair of the jaw bone could complete within 3–9 months, so the degradation rate of the composite scaffold had better match the growth rate of bone tissue cells in the repair area. Previous studies on nHA/GO materials have found that when the GO ratio is 0.1–1 wt%, the degradation time is prolonged, which is similar to the bone repair cycle.^43^ In this study, the degradation rates of nHA/FG composite scaffold were relatively slow, especially in the group of 1 wt%, the degradation rate was only (57.32 ± 1.40)% at the third month, while that of the nHA scaffold was (81.67 ± 3.13)%, proving that FG can slow down the degradation rate of scaffold materials. In the first 3 months, the nHA/FG composite scaffolds can still play a part of supporting role, the degradation time of scaffolds matches the bone growth cycle, which is expected to realize the synchronization of composite scaffold degradation and bone defect repair.

Due to the structural instability of nHA scaffold in aqueous solution, it has a certain degree of degradability and can continuously release Ca ion, P ion and FG, while the release of F ion is synchronous with the release of FG. The results of this study also confirmed that the nHA/FG composite scaffolds could slowly release Ca, P and F ions, and the released amounts of these substances increased by time, and the release rates were gradually decreased with the extension of time. This is consistent with the studies of previous studies.^26^ The ion release generally show short initial burst release followed by longer continuous but declining release, and the release pattern depends on the FG load of the scaffold material. In addition, the release of Ca and P ion decreased significantly with the increased content of FG. This is due to the degradation rate of composite scaffolds decreased with the increased FG content. However, the released amount of F ion increased with the increased content of FG, because there are not only more FG sources, but also a large number of F ions adsorbed or encapsulated by them are released. Moreover, the 1 wt% group of the nHA/FG composite scaffold released 0.3 mg L^−1^ at 6th months, which still had good cytocompatibility.

To ensure the biocompatibility of the material, the tissue engineered bone scaffold must be tested for cytocompatibility and hemocompatibility before implantation *in vivo*. MC3T3-E1 cells have good proliferation and differentiation potential *in vivo* and *in vitro*, and are often used as seed cells in bone tissue engineering research. Moreover, the time-dependence and expression of MC3T3-E1 cells *in vitro* culture are similar to those *in vivo*, so it is widely used to study the cytocompatibility of bone scaffolds. In this study, the CCK-8 method was used to detect the cytocompatibility, and the hemolysis rate was used to test the hemocompatibility of the composite scaffold. The results showed that the HA/FG composite scaffold had excellent cytocompatibility without hemolysis reaction. The fabricated composite scaffold can provide a suitable environment for cell growth and proliferation. Moreover, the products of simulated *in vitro* degradation of the composite scaffold material will not cause damage to blood in the short term. These will lay a good experimental foundation for the application of composite scaffolds in animals and humans beings.

One of the main reasons for the failure of bone defect repair is bacterial infection. After getting into the tissue, the bacteria will colonize and propagate, and then form a bacterial biofilm that are difficult to eradicate. Common pathogenic bacteria in bone infection are Gram-negative *E. coli* and Gram-positive *S. aureus*, these two kinds of bacterial strains were used in this experiment. The FG has antibacterial properties and it can be dispersed uniformly in the material as an antibacterial material. FG is readily available and without drug resistance. The mechanism of the antibacterial effect of FG mainly includes the destruction of the bacterial cell membrane by the sharp edge of FG, and the chemical damage caused by the oxidative stress of the lipid molecules in the bacterial cell membrane by fluoride ions. Previous studies have shown that fluorine ions can exert antibacterial effects by inhibiting the growth, reproduction and metabolism of bacteria and interfering with the formation of bacterial biofilms.^44^ In the present experiments, the nHA/FG composite scaffold modified with low concentration of FG was found to have good antibacterial properties. This is due to the surface of the scaffold contains FG, and there is also degraded FG in the surrounding environment. In addition, the antibacterial activity of FG against Gram-negative bacteria is higher than that against Gram-positive bacteria because the cell wall of Gram-negative bacteria is thinner than that of Gram-positive bacteria.^45^ However, the long-term antibacterial effect of the nHA/FG composite scaffold needs to be further investigated in future experiments.

## Conclusions

In this study, a simple hydrothermal method was used to prepare FG. The synthesized FG is a transparent nanostructure and is well dispersed in the nHA scaffold. Compared with the nHA scaffolds, the nHA/FG composite scaffolds exhibited better hydrophilicity and mechanical properties. Additionally, 0.5 wt% is considered to be an ideal addition with good biocompatibility and antibacterial activity. In summary, the nHA/FG composite scaffolds have the potential to reduce the risk of bacterial infections in bone tissue engineering.

## Ethical statement

All experiments were performed in accordance with the World Medical Association Declaration of Helsinki guidelines, and approved by the ethics committee of the Affiliated Hospital of Qingdao University. Informed consents were obtained from human participants of this study.

## Author contributions

Conceptualization and methodology, ZX; investigation, DX; resources, YX and YL; data curation, LL; writing—original draft preparation, ZX; writing—review and editing, YL; supervision, LC; project administration, JS; funding acquisition, JS. All authors have read and agreed to the published version of the manuscript.

## Conflicts of interest

There are no conflicts to declare.

## Supplementary Material

## References

[cit1] Deng N., Sun J., Li Y., Chen L., Chen C., Wu Y., Wang Z., Li L. (2019). Experimental study of rhBMP-2 chitosan nano-sustained release carrier-loaded PLGA/nHA scaffolds to construct mandibular tissue-engineered bone. Arch. Oral Biol..

[cit2] Riester O., Borgolte M., Csuk R., Deigner H. P. (2021). Challenges in bone tissue regeneration: stem cell therapy, biofunctionality and antimicrobial properties of novel materials and its evolution. Int. J. Mol. Sci..

[cit3] Sangkert S., Kamolmatyakul S., Meesane J. (2020). The bone-mimicking effect of calcium phosphate on composite chitosan scaffolds in maxillofacial bone tissue engineering. J. Appl. Biomater. Funct. Mater..

[cit4] Cui L., Zhang J., Zou J., Yang X., Guo H., Tian H., Zhang P., Wang Y., Zhang N., Zhuang X. (2020). *et al.*, Electroactive composite scaffold with locally expressed osteoinductive factor for synergistic bone repair upon electrical stimulation. Biomaterials.

[cit5] Liu F., Kang H., Liu Z., Jin S., Yan G., Sun Y., Li F., Zhan H., Gu Y. (2021). 3D printed multi-functional scaffolds based on poly(ε-caprolactone) and hydroxyapatite composites. Nanomaterials.

[cit6] Fu Z., Cui J., Zhao B., Shen S. G., Lin K. (2021). An overview of polyester/hydroxyapatite composites for bone tissue repairing. J. Orthop. Transl..

[cit7] Gao X., Wang H., Zhang X., Gu X., Liu Y., Zhou G., Luan S. (2020). Preparation of Amorphous Poly(aryl ether nitrile ketone) and Its Composites with Nano Hydroxyapatite for 3D Artificial Bone Printing. ACS Appl. Bio Mater..

[cit8] Kim J., Kang I. G., Cheon K. H., Lee S., Park S., Kim H. E., Han C. M. (2021). Stable sol–gel hydroxyapatite coating on zirconia dental implant for improved osseointegration. J. Mater. Sci.: Mater. Med..

[cit9] Mahato A., Sandy Z., Bysakh S., Hupa L., Das I., Bhattacharjee P., Kundu B., De G., Nandi S. K., Vallittu P. (2020). *et al.*, Development of nano-porous hydroxyapatite coated e-glass for potential bone-tissue engineering application: an *in vitro* approach. Mater. Sci. Eng., C.

[cit10] Benedini L., Laiuppa J., Santillán G., Baldini M., Messina P. (2020). Antibacterial alginate/nano-hydroxyapatite composites for bone tissue engineering: assessment of their bioactivity, biocompatibility, and antibacterial activity. Mater. Sci. Eng., C.

[cit11] Prasanna A. P. S., Venkatasubbu G. D. (2018). Sustained release of amoxicillin from hydroxyapatite nanocomposite for bone infections. Prog. Biomater..

[cit12] Eskandarinezhad M., Ghodrati M., Pournaghi Azar F., Jafari F., Samadi Pakchin P., Abdollahi A. A., Sadrhaghighi A. H., Rezvan F. (2020). Effect of Incorporating Hydroxyapatite and Zinc Oxide Nanoparticles on the Compressive Strength of White Mineral Trioxide Aggregate. J. Dent..

[cit13] Li D., Chen M., Guo W., Li P., Wang H., Ding W., Li M., Xu Y. (2022). In situ Grown Nanohydroxyapatite Hybridized Graphene Oxide: Enhancing the Strength and Bioactivity of Polymer Scaffolds. ACS Omega.

[cit14] Li M., Xiong P., Yan F., Li S., Ren C., Yin Z., Li A., Li H., Ji X., Zheng Y. (2018). *et al.*, An overview of graphene-based hydroxyapatite composites for orthopedic applications. Bioact. Mater..

[cit15] Maleki-Ghaleh H., Siadati M. H., Fallah A., Koc B., Kavanlouei M., Khademi-Azandehi P., Moradpur-Tari E., Omidi Y., Barar J., Beygi-Khosrowshahi Y. (2021). *et al.*, Antibacterial and cellular behaviors of novel zinc-doped hydroxyapatite/graphene nanocomposite for bone tissue engineering. Int. J. Mol. Sci..

[cit16] Gupta R. K., Malviya M., Verma C., Quraishi M. A. (2017). Aminoazobenzene and diaminoazobenzene functionalized graphene oxides as novel class of corrosion inhibitors for mild steel: experimental and DFT studies. Mater. Chem. Phys..

[cit17] Ramezanzadeh B., Kardar P., Bahlakeh G., Hayatgheib Y., Mahdavian M. (2017). Fabrication of a Highly Tunable Graphene Oxide Composite through Layer-by-Layer Assembly of Highly Crystalline Polyaniline Nanofibers and Green Corrosion Inhibitors: Complementary Experimental and First-Principles Quantum-Mechanics Modeling Approaches. J. Phys. Chem. C.

[cit18] Shahrouzifar M. R., Salahinejad E., Sharifi E. (2019). Co-incorporation of strontium and fluorine into diopside scaffolds: bioactivity, biodegradation and cytocompatibility evaluations. Mater. Sci. Eng., C.

[cit19] Shin D. Y., Cheon K. H., Song E. H., Seong Y. J., Park J. U., Kim H. E., Jeong S. H. (2019). Fluorine-ion-releasing injectable alginate nanocomposite hydrogel for enhanced bioactivity and antibacterial property. Int. J. Biol. Macromol..

[cit20] Romero-Aburto R., Narayanan T. N., Nagaoka Y., Hasumura T., Mitcham T. M., Fukuda T., Cox P. J., Bouchard R. R., Maekawa T., Kumar D. S. (2013). *et al.*, Fluorinated graphene oxide; a new multimodal material for biological applications. Adv. Mater..

[cit21] Chen X., Fan K., Liu Y., Li Y., Liu X., Feng W., Wang X. (2022). Recent Advances in Fluorinated Graphene from Synthesis to Applications: Critical Review on Functional Chemistry and Structure Engineering. Adv. Mater..

[cit22] Prasanthi I., Raidongia K., Datta K. K. R. (2021). Super-wetting properties of functionalized fluorinated graphene and its application in oil-water and emulsion separation. Mater. Chem. Front..

[cit23] Zhao F. G., Zhao G., Liu X. H., Ge C. W., Wang J. T., Li B. L., Wang Q. G., Li W. S., Chen Q. Y. (2014). Fluorinated graphene: facile solution preparation and tailorable properties by fluorine-content tuning. J. Mater. Chem. A.

[cit24] Liu R., Wang E., Guo Y., Zhou Q., Zheng Y., Zhai J., Zhang K., Zhang B. (2021). Enhanced antibacterial properties and promoted cell proliferation in glass ionomer cement by modified with fluorinated graphene-doped. J. Appl. Biomater. Funct. Mater..

[cit25] Xu L., Zheng Y., Yan Z., Zhang W., Shi J., Zhou F., Zhang X., Wang J., Zhang J., Liu B. (2016). Preparation, tribological properties and biocompatibility of fluorinated graphene/ultrahigh molecular weight polyethylene composite materials. Appl. Surf. Sci..

[cit26] Sun L., Yan Z., Duan Y., Zhang J., Liu B. (2018). Improvement of the mechanical, tribological and antibacterial properties of glass ionomer cements by fluorinated graphene. Dent. Mater..

[cit27] Colucci F., Mancini V., Mattu C., Boffito M. (2021). Designing multifunctional devices for regenerative pharmacology based on 3d scaffolds, drug-loaded nanoparticles, and thermosensitive hydrogels: a proof-of-concept study. Pharmaceutics.

[cit28] Yang Y., Xu Z., Guo Y., Zhang H., Qiu Y., Li J., Ma D., Li Z., Zhen P., Liu B. (2021). *et al.*, Novel core–shell CHX/ACP nanoparticles effectively improve the mechanical, antibacterial and remineralized properties of the dental resin composite. Dent. Mater..

[cit29] Seyfert U. T., Biehl V., Schenk J. (2002). In vitro hemocompatibility testing of biomaterials according to the ISO 10993-4. Biomol. Eng..

[cit30] Zhou D., Cheng L., Xu D., Xu Z., Sun M., Chen L., Liu Y., Sun J. (2022). Formulation and performance of bioactive hydrogel scaffold carrying chlorhexidine and bone morphogenetic protein. Mater. Lett..

[cit31] Datta K. K. R. and Zbořil R., Halogenated Graphenes: Emerging Family of Two-Dimensional Materials, Functionalization of Graphene, 2014, pp. 173–198

[cit32] Wang W., Xu Z., Zhang X., Wimmer A., Shi E., Qin Y., Zhao X., Zhou B., Li L. (2018). Rapid and efficient removal of organic micropollutants from environmental water using a magnetic nanoparticles-attached fluorographene-based sorbent. Chem. Eng. J..

[cit33] Herraiz M., Dubois M., Batisse N., Hajjar-Garreau S., Simon L. (2018). Large-scale synthesis of fluorinated graphene by rapid thermal exfoliation of highly fluorinated graphite. Dalton Trans..

[cit34] Min C., He Z., Liang H., Liu D., Dong C., Song H., Huang Y. (2020). High mechanical and tribological performance of polyimide nanocomposite reinforced by fluorinated graphene oxide. Polym. Compos..

[cit35] Krathumkhet N., Sabrina, Imae T., Krafft M. P. (2021). Nitric Oxide Gas in Carbon Nanohorn/Fluorinated Dendrimer/Fluorinated Poly(ethylene glycol)-Based Hierarchical Nanocomposites as Therapeutic Nanocarriers. ACS Appl. Bio Mater..

[cit36] Gong P., Zhao Q., Dai D., Zhang S., Tian Z., Sun L., Ren J., Liu Z. (2017). Functionalized Ultrasmall Fluorinated Graphene with High NIR Absorbance for Controlled Delivery of Mixed Anticancer Drugs. Chem.–Eur. J..

[cit37] Xie C., Gao Q., Wang P., Shao L., Yuan H., Fu J., Chen W., He Y. (2019). Structure-induced cell growth by 3D printing of heterogeneous scaffolds with ultrafine fibers. Mater. Des..

[cit38] Lai W., Xu D., Wang X., Wang Z., Liu Y., Zhang X., Liu X. (2017). Characterization of the thermal/thermal oxidative stability of fluorinated graphene with various structures. Phys. Chem. Chem. Phys..

[cit39] Ren M., Wang X., Dong C., Li B., Liu Y., Chen T., Wu P., Cheng Z., Liu X. (2015). Reduction and transformation of fluorinated graphene induced by ultraviolet irradiation. Phys. Chem. Chem. Phys..

[cit40] Meng S., Fu X., Jiang L., Shi L., Wang X., Liu X., Wang J. (2022). Theoretical calculations and experiments on the thermal properties of fluorinated graphene and its effects on the thermal decomposition of nitrate esters. Nanomaterials.

[cit41] Peng S., Feng P., Wu P., Huang W., Yang Y., Guo W., Gao C., Shuai C. (2017). Graphene oxide as an interface phase between polyetheretherketone and hydroxyapatite for tissue engineering scaffolds. Sci. Rep..

[cit42] Babilotte J., Martin B., Guduric V., Bareille R., Agniel R., Roques S., Héroguez V., Dussauze M., Gaudon M., Le Nihouannen D. (2021). *et al.*, Development and characterization of a PLGA-HA composite material to fabricate 3D-printed scaffolds for bone tissue engineering. Mater. Sci. Eng., C.

[cit43] Llorens E., Calderón S., Del Valle L. J., Puiggalí J. (2015). Polybiguanide (PHMB) loaded in PLA scaffolds displaying high hydrophobic, biocompatibility and antibacterial properties. Mater. Sci. Eng., C.

[cit44] Geng H., Wang T., Cao H., Zhu H., Di Z., Liu X. (2019). Antibacterial ability, cytocompatibility and hemocompatibility of fluorinated graphene. Colloids Surf., B.

[cit45] Pan F., Altenried S., Zuber F., Wagner R. S., Su Y. H., Rottmar M., Maniura-Weber K., Ren Q. (2021). Photo-activated titanium surface confers time dependent bactericidal activity towards Gram positive and negative bacteria. Colloids Surf., B.

